# A Successful Matinee

**Published:** 1913-12-20

**Authors:** 


### A Successful Matinee.

What can be done in support of our hospitals by an enthusiastic committee is disclosed in the balance sheet of the Leicester Royal Infirmary Matinée Committee as the result of a matinée at the Palace Theatre, which was most kindly placed at the disposal of the committee by Mr. Oswald Stoll. The total receipts amount to £202 6s. 2d., and as Mr. Oswald Stoll most kindly arranged the whole programme free of cost to the committee, and the expenses of printing, posters, etc., were borne by the chairman of the matinée committee (Mr. H. H. Wildt), it has been possible to hand over the entire sum to the institution. This successful organisation of the matinée by Mr. H. H. Wildt, Mr. T. H. Prentice (Vice-Chairman), Mr. A. Baum (Treasurer), Mr. H. Thomsett (Honorary Auditor), and Mr. G. F. Reynolds and Mr. J. H. Ward, Honorary Secretaries, has given great encouragement to the Board of Governors of the infirmary.

